# Battling Right Ventricular Dysfunction in Post-Infarction Ventricular Septal Defect—A Case Report and Comprehensive Review of Literature

**DOI:** 10.3390/life16050808

**Published:** 2026-05-12

**Authors:** Horatiu Moldovan, Irina Dobra, Sabina Safta, Mircea Robu, Andrada Guta, Silvia Preda, Alexandra Voicu, Maria Girel, Alexandru Alexandrescu, Ondin Zaharia

**Affiliations:** 1 Faculty of Medicine, “Carol Davila” University of Medicine and Pharmacy, 050474 Bucharest, Romania; horatiu.moldovan@umfcd.ro (H.M.);; 2 Academy of Romanian Scientists, 050711 Bucharest, Romania; 3 Department of Cardiovascular Surgery, Clinical Emergency Hospital Bucharest, 014461 Bucharest, Romaniaalexandru.alexandrescu@yahoo.com (A.A.)

**Keywords:** post-infarction ventricular septal defect, surgical repair timing, cardiogenic shock, temporary mechanical life support

## Abstract

Post-infarction ventricular septal defect (VSD) represents a rare but frequently fatal mechanical complication of ST-elevation myocardial infarction (STEMI), associated with high morbidity and mortality despite advances in reperfusion strategies. The optimal timing of surgical repair remains a matter of ongoing debate, particularly in patients presenting with hemodynamic instability and evolving right ventricular failure. Two main strategies have been proposed: an early surgical approach aimed at preventing progressive hemodynamic deterioration and right ventricular dysfunction, and a delayed strategy that allows for infarct maturation and fibrotic remodeling of the septal margins, thereby facilitating more secure patch anchoring and reducing the risk of residual shunting. We report the case of a 39-year-old male with multiple cardiovascular risk factors who presented to the emergency department after seven days of persistent chest pain and was diagnosed with an inferior STEMI. Urgent percutaneous coronary intervention was performed, with successful stent implantation in the right coronary artery. Seven days later, transthoracic echocardiography identified an inferior post-infarction ventricular septal defect. In the context of clinical deterioration characterized by progressive right ventricular failure, urgent surgical repair was undertaken. The postoperative course was complicated by severe pulmonary hypertension and refractory cardiogenic shock, necessitating veno-arterial extracorporeal membrane oxygenation (VA-ECMO) support for five days. The patient was subsequently weaned successfully from mechanical circulatory support and discharged on postoperative day 12. At one- and three-month follow-up, he remained asymptomatic, with significant recovery of left ventricular ejection fraction. This case underscores the critical importance of timely surgical intervention in post-infarction VSD, particularly in the setting of right ventricular failure, and highlights the essential role of temporary mechanical circulatory support in the management of severe postoperative cardiogenic shock.

## 1. Introduction

Although mechanical complications of acute myocardial infarction have become uncommon in the reperfusion era, myocardial rupture remains one of the most lethal events following STEMI. Myocardial rupture may be classified as external rupture, represented mainly by left ventricular free-wall rupture leading to hemopericardium and cardiac tamponade, or internal rupture, including ventricular septal rupture and papillary muscle rupture. Left ventricular free-wall rupture is generally considered the most frequent form of post-infarction myocardial rupture. Its incidence has decreased substantially after the widespread adoption of primary percutaneous coronary intervention, from values reported as high as approximately 6% in STEMI patients before the PCI era to approximately 0.01–0.5% in contemporary myocardial infarction cohorts [1].

Post-myocardial infarction ventricular septal defect (VSD) is a rare but catastrophic mechanical complication of acute myocardial infarction, typically occurring within the first week after the ischemic event [1,2]. Despite a significant decline in incidence in the reperfusion era, it remains associated with extremely high mortality, particularly in patients who rapidly develop cardiogenic shock [2,3]. Surgical repair represents the only definitive treatment; however, the optimal timing of intervention remains controversial [4].

From an anatomical perspective, post-infarction VSDs are broadly classified as anterior or posterior, depending on infarct location. Anterior defects usually involve the apical septum following left anterior descending artery occlusion, whereas posterior defects complicate inferior infarction and often involve the basal inferoposterior septum [1,5]. Posterior defects are associated with more complex surgical exposure and are frequently linked to worse clinical outcomes [5,6].

Hemodynamic deterioration is driven by the abrupt onset of a left-to-right shunt, resulting in pulmonary overcirculation and reduced effective systemic output [1,2]. Right ventricular dysfunction is a key determinant of prognosis, particularly in inferior infarction, where direct ischemic injury of the right ventricle may coexist with acute volume overload and increased pulmonary pressures, leading to progressive right-sided failure [1,6].

Two main strategies have been proposed regarding the timing of surgical repair. An early intervention approach aims to prevent ongoing hemodynamic deterioration and right ventricular failure, whereas a delayed strategy allows for infarct maturation and fibrosis of the septal margins, facilitating more secure patch fixation and potentially reducing the risk of residual shunt [4,7]. In clinical practice, the timing of surgery is often dictated by the patient’s hemodynamic status and the feasibility of temporary stabilization [4].

Surgical repair typically involves exclusion of the infarcted myocardium and closure of the defect using a patch, most commonly bovine pericardium, with the goal of minimizing residual shunting while preserving ventricular geometry [8]. In this setting, mechanical circulatory support has emerged as an important adjunct, either as a bridge to surgery or for the management of postoperative cardiogenic shock [9,10].

## 2. Case Presentation

A 39-year-old male patient presented at the territorial hospital with chest pain. The symptomatology started 7 days earlier. Given the young age of the patient, the cardiovascular risk profile was further detailed. In addition to obesity, active heavy smoking, arterial hypertension, and dyslipidemia, the patient had a significant paternal family history of premature ischemic heart disease; his father had documented coronary artery disease requiring multiple coronary stent implantations beginning at the age of 45 years. The baseline lipid profile showed marked dyslipidemia, with total cholesterol of 320 mg/dL, LDL-cholesterol of 160 mg/dL, triglycerides of 189 mg/dL, and HDL-cholesterol of 15 mg/dL.

The admission electrocardiogram was consistent with inferior STEMI, showing ST-segment elevation in leads II, III, and aVF, with reciprocal ST-segment depression in leads I and aVL. Postoperatively, the electrocardiogram showed sinus rhythm, complete left bundle branch block, and pathological Q waves in the inferior leads, consistent with established inferior myocardial necrosis.

He was diagnosed with inferior ST-elevated myocardial infarction and emergency coronary angiography with stent implantation on the right coronary artery was performed. Also, a severe 70% stenosis in the first segment of the left anterior descending artery was diagnosed.

**Figure 1:**
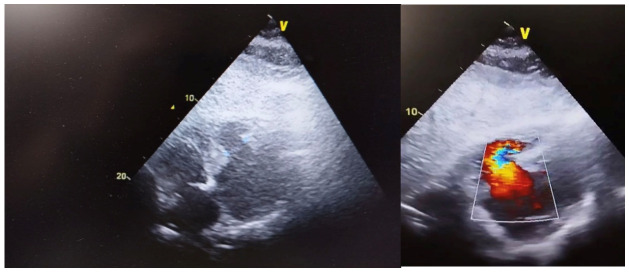
Echocardiography showing left-to-right shunt with color gradient.

Within the early post-infarction period, the patient developed acute hemodynamic deterioration characterized by hypotension and a new harsh holosystolic murmur. Transthoracic echocardiography revealed a large inferior ventricular septal defect with significant left-to-right shunting and preserved initial left ventricular systolic function. Thus, the patient was transferred from the territorial hospital to our department and admitted to the ICU for monitoring and close-up evaluation. On transoesophageal echocardiography, the large inferior ventricular septal defect of circa 28 mm with left-to-right shunt was seen from both transoesophageal view at 30 degrees axis and transgastric view. On colour Doppler exam, a gradient of circa 9 mmHg was suggestive of a ventricular septal defect, as seen in Figure 1.

Due to rapidly worsening right ventricular dysfunction and progressive cardiogenic shock, urgent surgical repair was decided within the first 24 h from patient admission in our department.

Preoperatively, the patient exhibited a rapidly progressive hemodynamic profile consistent with cardiogenic shock predominantly driven by right ventricular dysfunction in the setting of an inferior post-infarction VSD. Invasive monitoring using a Swan–Ganz pulmonary artery catheter demonstrated markedly elevated right-sided and pulmonary pressures, with a mean pulmonary artery pressure of 42 mmHg, systolic pulmonary artery pressure of 73 mmHg, central venous pressure of 21 mmHg, and pulmonary capillary wedge pressure of 19 mmHg. Cardiac output and cardiac index were severely reduced, measuring 3.1 L/min and 1.8 L/min/m^2^, respectively, consistent with low-output cardiogenic shock. Echocardiography confirmed severe right ventricular dysfunction, with right ventricular dilatation, reduced radial and longitudinal contractility, tricuspid annular plane systolic excursion of 12 mm and fractional area change of 28%. Estimated systolic pulmonary artery pressure on echocardiography was 68 mmHg, supporting the presence of severe pulmonary hypertension. Serial arterial lactate measurements showed progressive metabolic deterioration, increasing from 2.1 mmol/L to 4.2 mmol/L and subsequently to 5.2 mmol/L over three consecutive hourly assessments, despite escalating pharmacological support. At the same time, the patient became anuric, indicating severe systemic hypoperfusion and evolving end-organ dysfunction. Taken together, these findings supported the decision to proceed with urgent surgical repair, as further temporization was considered unsafe in the context of progressive right ventricular failure, severe pulmonary hypertension, low cardiac output, rising lactate, and absence of urine output.

**Figure 2:**
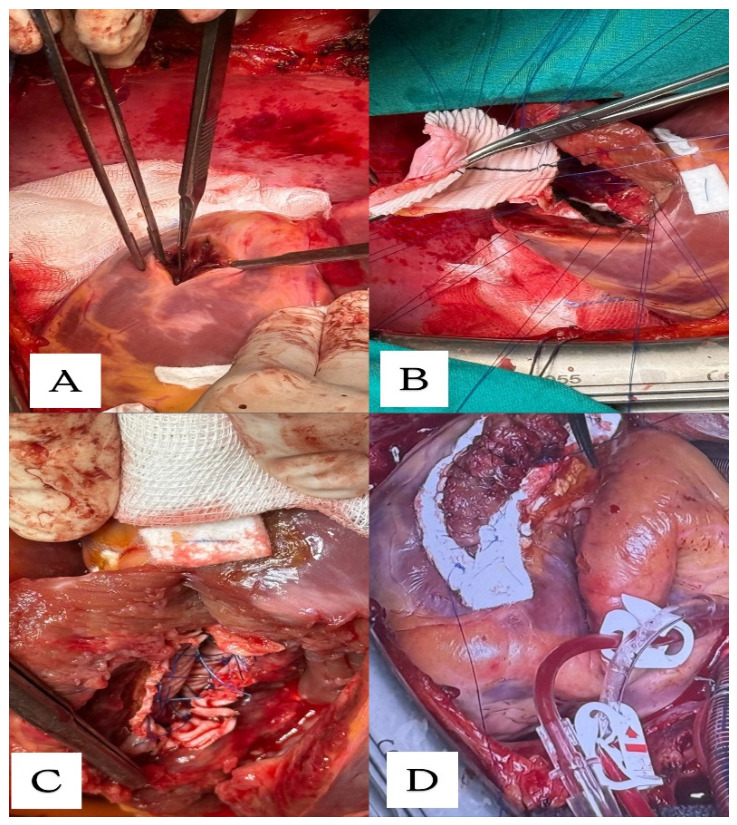
Intraoperative images: (**A**) Left ventriculotomy in infarction zone and parallel to the posterior interventricular groove; (**B**) Tailored composite pericardium–Dacron patch; (**C**) Final patch placement; (**D**) Linear closure on Teflon strips of ventriculotomy.

The surgical approach was via median sternotomy and on initial direct inspection the patient had dilatated right cavities, a dilatated pulmonary artery and a right ventricle with diminished contractility. Central bi-caval cannulation and anterograde cardioplegia were administered. On inferior wall inspection the infarction zone contained both right and left ventricles. Left ventriculotomy was done through the infarction zone and parallel to the posterior interventricular groove (Figure 2A). Thus, the interventricular septal defect was exposed. The defect was complete and the surgeon could pass 2 fingers through it. The reconstruction of the defect was achieved with a tailored composite patch of an autologous pericardial patch with a synthetic Dacron fabric patch sutured to the endocardium of the left ventricle with discontinuous circular Prolene 3.0 stiches (Figure 2B). The ventriculotomy was closed in two layers of sutures buttressed with strips of Teflon (Figure 2D). Furthermore, after the defect repair complete myocardial revascularization was achieved with a bypass of the left internal mammary artery to the left anterior descending artery. Intraoperative transoesophageal echocardiography confirmed an adequate repair of the interventricular septum and the patient was transferred successfully to the ICU unit.

In the immediate postoperative period, despite adequate anatomical closure of the ventricular septal defect and absence of significant residual shunting on transesophageal echocardiography, the patient developed refractory cardiogenic shock due to severe biventricular dysfunction, with predominant right ventricular failure. Echocardiography demonstrated severely impaired right ventricular systolic function, persistent right ventricular dilatation, reduced longitudinal shortening with TAPSE of 11 mm, reduced fractional area change of 26%, and impaired left ventricular systolic performance with an estimated left ventricular ejection fraction of 15%. Invasive Swan–Ganz monitoring showed persistent pulmonary hypertension, with systolic pulmonary artery pressure of 81 mmHg, mean pulmonary artery pressure of 29 mmHg, central venous pressure of 23 mmHg, and a reduced cardiac index of 1.2 L/min/m^2^. Serum lactate continued to rise postoperatively, reaching 4.2 mmol/L, 5.2 mmol/L, and subsequently 6.1 mmol/L on serial hourly measurements, while urine output remained absent despite optimization of preload, ventilation, and pharmacological support. Vasoactive and inotropic requirements progressively increased from moderate to high doses, including norepinephrine from 200 μg/kg/min to 600 μg/kg/min, dobutamine from 3 μg/kg/min to 10 μg/kg/min, and epinephrine from 70 ng/kg/min to 250 ng/kg/min. This combination of persistent pulmonary hypertension, severe biventricular dysfunction, rising lactate, anuria, and escalating vasoactive support fulfilled the criteria for refractory postcardiotomy cardiogenic shock and prompted urgent initiation of peripheral VA-ECMO.

Peripheral VA ECMO was initiated as rescue mechanical circulatory support and was maintained for five days. Initial ECMO blood flow was set between 4.0 and 5.0 L/min, then adjusted according to systemic perfusion, lactate clearance, arterial pressure, pulmonary pressures, and serial echocardiographic assessment. Particular attention was paid to maintaining aortic and mitral valve opening in order to avoid excessive left ventricular distension and pulmonary congestion. Following VA-ECMO initiation, systemic perfusion improved rapidly, allowing a significant reduction in vasoactive and inotropic support. Norepinephrine was decreased to 120 μg/kg/min, and dobutamine to 4.5 μg/kg/min over the subsequent 7 h. Serum lactate reached a maximum of 9.0 mmol/L, followed by progressive clearance and normalization. Urine output gradually recovered after restoration of systemic perfusion. The patient also developed transient hepatic and renal dysfunction, with peak serum transaminases of AST 258 U/L and ALT 429 U/L, serum urea of 138 mg/dL, and serum creatinine of 2.5 mg/dL, all of which progressively improved during circulatory support and after successful VA—ECMO weaning. Peripheral VA-ECMO weaning was performed under serial echocardiographic and hemodynamic guidance after progressive improvement in biventricular function, normalization of lactate, recovery of urine output, and reduction in vasoactive support to low-dose requirements. Levosimendan was administered before weaning as an inodilator strategy to improve myocardial contractility while limiting further increases in ventricular afterload. During stepwise VA—ECMO flow reduction, echocardiography confirmed preserved aortic valve opening, absence of left ventricular distension, no significant residual interventricular shunt, improved right ventricular contractility, and stable left ventricular systolic performance. Hemodynamic stability was maintained during low-flow testing, with adequate mean arterial pressure, acceptable pulmonary artery pressures, stable cardiac index, and no recurrent lactate elevation. Based on these findings, the patient was successfully decannulated without major complications. The patient showed continuous clinical improvement thereafter and was discharged on the 12th postoperative day under optimized guideline-directed medical therapy.

At discharge, the patient received optimized guideline-directed medical therapy for secondary prevention after STEMI and for heart failure with reduced systolic function. The regimen included dual antiplatelet therapy, high-intensity statin therapy, beta-blocker, renin–angiotensin system inhibition or angiotensin receptor–neprilysin inhibition as tolerated, mineralocorticoid receptor antagonist, sodium–glucose cotransporter 2 inhibitor, and loop diuretic therapy according to congestion status, together with smoking cessation counseling, dietary intervention, and cardiac rehabilitation. At three-month follow-up, the patient remained asymptomatic, without clinical signs of heart failure, and LDL-cholesterol had decreased to 120 mg/dL under statin therapy and dietary measures.

**Figure 3:**
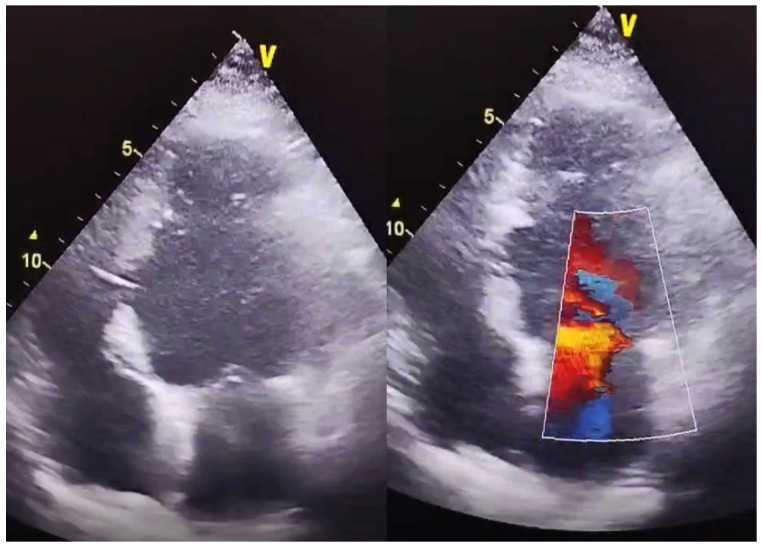
Postoperative follow-up echocardiography with no residual shunt.

Transthoracic echocardiography demonstrated complete closure of the ventricular septal defect and an improved ejection fraction, as shown in Figure 3.

## 3. Review

Post-myocardial infarction ventricular septal defect remains a rare but catastrophic mechanical complication of acute myocardial infarction, most commonly occurring within the first week after the ischemic event [1,2]. Although its incidence has declined in the reperfusion era, mortality remains high, particularly in patients who develop cardiogenic shock or multiorgan hypoperfusion [1,3]. Anatomically, post-infarction VSDs are usually classified as anterior or posterior according to infarct location and the involved coronary territory [1,6]. Anterior defects generally follow extensive anterior myocardial infarction and involve the apical or anteroapical septum, whereas posterior defects typically complicate inferior infarction and involve the basal inferoposterior septum [5,6]. Posterior defects are more technically challenging because of their deeper location, friable surrounding myocardium, complex three-dimensional geometry, and difficult patch anchoring [5,6]. These anatomical features may explain the worse outcomes reported in surgical series and registry-based analyses, including higher operative mortality and increased rates of residual shunting compared with anterior lesions [4,5,6].

Hemodynamic deterioration is driven by the abrupt establishment of a left-to-right shunt, resulting in pulmonary overcirculation and reduced effective systemic cardiac output. The severity of systemic hypoperfusion has major prognostic implications. In the classic hemodynamic analysis by Moore et al., survivors had a significantly higher effective cardiac index than nonsurvivors, whereas cardiogenic shock was associated with mortality rates exceeding 90% [1]. Right ventricular dysfunction represents a particularly important determinant of outcome in inferior or posterior infarction, where right ventricular ischemic involvement may coexist with acute shunt-related volume overload [2]. The combination of increased right ventricular preload, elevated pulmonary blood flow, secondary pulmonary hypertension, and increased right ventricular afterload may create a rapidly progressive cycle of right-sided failure. Elevated right atrial pressure, previously identified as an independent predictor of mortality, reflects advanced right ventricular failure and correlates with impaired systemic perfusion [3]. Ventricular interdependence, septal shift, and impaired left ventricular filling may further amplify the decline in cardiac output and contribute to end-organ dysfunction. Thus, in post-infarction VSD, right ventricular dysfunction should be regarded as a multifactorial process resulting from infarct topography, acute shunt physiology, and secondary pulmonary vascular burden.

Surgical repair remains the reference treatment for post-infarction VSD because it provides definitive exclusion of the rupture and allows management of necrotic myocardium. Nevertheless, operative mortality remains substantial, particularly in patients presenting with cardiogenic shock, right ventricular dysfunction, or posterior defects [5]. Surgery is generally preferred in patients with large or complex defects, posterior or basal ruptures, extensive myocardial necrosis, or anatomy unsuitable for device closure, and remains the most reliable method for minimizing residual shunting [4,5]. Contemporary surgical repair is based primarily on infarct exclusion and patch closure of the septal rupture, most commonly using biologic or synthetic materials. The infarct-exclusion technique popularized by David and colleagues avoids placing sutures directly into friable necrotic tissue and remains a cornerstone of modern surgical management [6]. Other strategies, including single-patch, double-patch, and tailored reconstructions, have been described according to defect size, location, and the extent of myocardial destruction [4,11,12]. These principles are particularly important in posterior or inferobasal defects, where complex exposure, proximity to the mitral valve apparatus, and fragile tissue increase the risk of residual shunting, patch dehiscence, and technical failure [4,5].

Percutaneous closure has emerged as an alternative strategy in selected patients considered at prohibitive surgical risk, as definitive therapy in carefully selected cases, as a bridge to delayed surgery, or for residual or recurrent defects after surgical repair [13]. The procedure is usually performed under fluoroscopic and transesophageal echocardiographic guidance, often using double-disc occluder devices such as the Amplatzer post-infarction VSD occluder or atrial septal defect occluders adapted for this indication [13,14,15]. However, procedural success depends heavily on defect morphology, size, and the presence of adequate septal rims, which are often absent in the acute phase because of friable necrotic tissue. Device instability, malposition, embolization, residual shunting, hemolysis, arrhythmias, vascular complications, and the need for repeat intervention remain important limitations [14,16]. Direct comparisons between surgical and percutaneous strategies remain limited by selection bias; although some analyses suggest lower in-hospital mortality after surgical repair, long-term mortality may not differ significantly, while transcatheter closure is often associated with higher rates of residual shunt and reintervention [13,16,17].

Timing remains one of the most debated aspects of management. Early intervention may prevent progressive hemodynamic deterioration and right ventricular failure, but is associated with increased procedural risk because of friable infarcted tissue. Conversely, delayed repair allows infarct maturation and fibrosis of the septal margins, potentially facilitating more secure patch fixation and reducing residual shunting or patch dehiscence. In unstable patients with cardiogenic shock, severe heart failure, progressive right ventricular dysfunction, or multiorgan hypoperfusion, conservative management or prolonged delay is associated with extremely poor outcomes, and urgent or emergent repair is recommended when hemodynamic stabilization cannot be achieved [2,3,18]. However, early surgery carries high operative risk, with registry data showing increased mortality when repair is performed within the first 7 days after myocardial infarction [5]. In contrast, delayed or temporized repair beyond 7–10 days has been associated with improved outcomes in selected stabilized patients, although this apparent benefit is influenced by selection bias because only patients who survive and can be stabilized are eligible for delayed intervention [5,17].

Mechanical circulatory support has increasingly reshaped this timing paradigm. Intra-aortic balloon pump, Impella, and veno-arterial extracorporeal membrane oxygenation may improve systemic perfusion, reduce hemodynamic instability, and serve either as a bridge to delayed repair or as support after definitive closure [9,19]. Therefore, the decision between early and delayed intervention should not be regarded as binary, but rather as an individualized Heart Team-based process integrating hemodynamic status, defect anatomy, ventricular function, end-organ perfusion, and the availability of mechanical circulatory support [17]. In patients with refractory cardiogenic shock, rapidly worsening right ventricular failure, or multiorgan dysfunction, immediate surgery may remain mandatory despite increased operative risk; conversely, in stable or stabilized patients, delayed repair may allow safer reconstruction under more favorable tissue conditions.

VA-ECMO has become an important adjunct in the management of post-infarction VSD, particularly in patients with refractory postcardiotomy shock after surgical closure [9,20]. Despite successful anatomical repair, persistent pulmonary hypertension, myocardial stunning, residual shunting, or biventricular dysfunction may maintain severe hemodynamic instability. VA-ECMO provides immediate circulatory and respiratory support, ensuring end-organ perfusion while allowing myocardial recovery [9,20]. Early initiation of mechanical circulatory support in postcardiotomy shock has been associated with improved survival compared with delayed escalation [20,21]. However, peripheral VA-ECMO may increase left ventricular afterload and contribute to ventricular distension, pulmonary edema, or impaired myocardial recovery, making adjunctive unloading strategies such as intra-aortic balloon pump, Impella, or surgical venting necessary in selected cases [22,23]. Reported survival in patients requiring VA-ECMO for postcardiotomy shock remains variable, generally ranging between 30% and 50%, with worse outcomes in those with delayed initiation, advanced age, or multiorgan failure [20,24]. Complications such as bleeding, limb ischemia, thromboembolic events, infection, hemolysis, ventricular distension, residual shunting, or patch dehiscence require careful monitoring and individualized adjustment of support [22,24]. In selected patients, VA-ECMO may serve either as a bridge to recovery after repair or as a bridge to delayed surgery by allowing temporary stabilization and infarct maturation under controlled hemodynamic conditions [19,21].

Beyond ventricular septal rupture, the present case should also be interpreted within the broader spectrum of post-infarction myocardial rupture syndromes. Myocardial rupture after acute myocardial infarction includes both external rupture, most commonly left ventricular free-wall rupture with hemopericardium and cardiac tamponade, and internal rupture, including ventricular septal rupture and papillary muscle rupture. Although all these entities have become less frequent in the reperfusion era, they remain among the most lethal mechanical complications of STEMI. Left ventricular free-wall rupture is generally considered the most frequent form of post-infarction myocardial rupture and may present either as an acute “blow-out” rupture with electromechanical dissociation and sudden death, or as a subacute/contained rupture with pseudoaneurysm formation and transient hemodynamic stabilization. Contemporary reports suggest that the incidence of left ventricular free-wall rupture has decreased to below 1% of myocardial infarction cases, but mortality remains extremely high, especially when diagnosis and surgical management are delayed [25,26]. In contrast, ventricular septal rupture represents an internal rupture phenotype in which the dominant pathophysiological mechanism is not tamponade but abrupt left-to-right shunting, pulmonary overcirculation, systemic hypoperfusion, and, particularly in inferior infarction, progressive right ventricular failure. This distinction is clinically important because both internal and external ruptures require rapid recognition and multidisciplinary management, but their hemodynamic profiles, imaging findings, and therapeutic priorities differ substantially.

In summary, post-infarction VSD remains a devastating complication in which prognosis is determined by defect anatomy, infarct location, shunt severity, ventricular function, and the ability to achieve timely hemodynamic stabilization. Surgical repair remains the cornerstone of definitive treatment, while percutaneous closure and mechanical circulatory support may play important roles in selected patients. No universal strategy is appropriate for all cases; rather, timing, technique, and adjunctive support should be tailored through a multidisciplinary Heart Team approach according to the patient’s evolving clinical profile.

## 4. Discussion

The present case highlights several key aspects in the contemporary management of post-infarction VSD, particularly the interplay between infarct topography, right ventricular dysfunction, and the timing of intervention. Our patient developed a large inferior VSD following delayed presentation of STEMI, a scenario well known to be associated with more complex anatomical defects and a higher incidence of right ventricular involvement. The rapid progression toward right ventricular failure observed in this case is consistent with previous reports demonstrating that inferior infarction frequently involves the right ventricle and leads to a more severe hemodynamic profile compared to anterior defects.

In this context, the decision to proceed with early surgical repair was driven by progressive hemodynamic deterioration and the inability to achieve adequate stabilization. Although delayed surgery is often associated with improved outcomes due to infarct maturation and more stable tissue for repair, this strategy is not feasible in patients with refractory cardiogenic shock or evolving right ventricular failure. The educational value of the present case lies not in the description of post-infarction VSD as an isolated mechanical complication, but in the dynamic interaction between inferior infarct location, right ventricular dysfunction, surgical timing, and postoperative mechanical circulatory support. While the benefits and limitations of early versus delayed repair have been widely discussed, this case illustrates that progressive right ventricular failure may become the dominant determinant of timing, making surgical temporization unsafe despite the known risks of operating on friable infarcted tissue. In inferior or posterior defects, right ventricular ischemic involvement may coexist with acute volume overload caused by the left-to-right shunt, increased pulmonary blood flow, secondary pulmonary hypertension, and ventricular interdependence, thereby creating a rapidly self-amplifying cycle of hemodynamic deterioration. In this context, the absence of residual shunt after repair does not necessarily equate to immediate hemodynamic recovery, as postoperative right ventricular failure and pulmonary hypertension may persist and precipitate refractory cardiogenic shock. The successful use of VA-ECMO in this patient therefore reinforces the concept that management should extend beyond anatomical closure of the defect and should include early recognition of right ventricular failure, timely escalation to mechanical circulatory support, and individualized Heart Team-based decision-making. Thus, this case provides a clinically relevant framework for unstable patients with inferior post-infarction VSD, in whom right ventricular dysfunction may represent the key factor linking early surgery to postoperative bridge-to-recovery support.

From a technical perspective, the use of an infarct-exclusion strategy with a composite pericardial–Dacron patch allowed effective closure of the defect while avoiding excessive tension on friable myocardial tissue. The inferior localization required a left ventriculotomy through the infarcted zone, a well-described but technically demanding approach, particularly in the setting of biventricular involvement. The absence of residual shunt on postoperative imaging confirms the effectiveness of this tailored surgical strategy.

In this case, the surgical strategy was selected according to the anatomical complexity and tissue characteristics of the defect. The VSD was large, inferior/posterior, and located within friable infarcted myocardium involving both ventricles; therefore, direct closure or a simple patch repair would have carried a substantial risk of suture tearing, residual shunting, and patch dehiscence. An infarct-exclusion technique was preferred because it allowed exclusion of the necrotic septal area from the high-pressure left ventricular cavity and permitted patch anchoring to relatively more viable endocardial tissue, thereby reducing tension on the repair. The use of a tailored composite autologous pericardial–Dacron patch was intended to combine the pliability and biological adaptability of pericardium with the mechanical strength of Dacron, providing a stable reconstruction for a large and irregular inferior defect. This approach was considered particularly appropriate for an inferior/posterior VSD, where surgical exposure is more difficult, the surrounding myocardium is often fragile, and the risk of residual shunting is higher than in anterior defects.

Concomitant myocardial revascularization was performed because coronary angiography had also identified a significant 70% stenosis of the proximal left anterior descending artery, segment I. This lesion was not treated during the initial percutaneous coronary intervention because the patient was critically unstable, and the immediate priority was rapid reperfusion of the culprit right coronary artery lesion. Once open surgical repair became necessary, LIMA-to-LAD bypass was performed to achieve complete revascularization, avoid leaving a prognostically important proximal LAD stenosis untreated, and reduce the risk of subsequent ischemic events. In addition, given the young age of the patient, the left internal mammary artery was considered the optimal conduit for durable LAD revascularization.

The postoperative course was marked by severe pulmonary hypertension and refractory cardiogenic shock, with right ventricular failure predominating. This evolution reflects the well-described pathophysiological cascade in post-infarction VSD, where abrupt changes in loading conditions, combined with pre-existing ischemic injury, lead to acute biventricular dysfunction. In this setting, the initiation of veno-arterial ECMO proved to be a life-saving intervention, providing immediate circulatory support and allowing time for right ventricular recovery.

The successful use of VA-ECMO in this case highlights its role as a bridge-to-recovery strategy in patients with postcardiotomy shock. However, it also illustrates the importance of careful hemodynamic management, as ECMO may increase left ventricular afterload and potentially worsen shunt dynamics if not appropriately balanced with adjunctive unloading strategies. The favorable outcome in our patient suggests that early initiation, combined with close monitoring and timely weaning, can significantly improve survival in this high-risk population.

Overall, this case reinforces the concept that post-infarction VSD should be approached as a dynamic and multifactorial condition, in which outcomes depend not only on defect closure but also on the management of ventricular dysfunction and systemic perfusion. The integration of surgical expertise, advanced imaging, and mechanical circulatory support is essential for optimizing patient outcomes. It is important to acknowledge that the evidence guiding management of post-infarction VSD remains limited and is largely based on retrospective series, registry data, observational studies, and meta-analyses affected by significant selection bias. Consequently, conclusions regarding the superiority of early versus delayed repair should be interpreted cautiously. Although urgent surgery is often required in patients with refractory cardiogenic shock, progressive right ventricular failure, or ongoing end-organ hypoperfusion, this does not imply that early repair is universally superior; rather, it reflects the fact that unstable patients frequently cannot tolerate temporization. Conversely, the better outcomes reported with delayed repair may partly reflect survival and selection bias, because only patients who can be stabilized long enough are eligible for postponement. Similarly, although mechanical circulatory support, including VA-ECMO, may provide temporary hemodynamic stabilization and allow myocardial or end-organ recovery, its impact on survival in post-infarction VSD has not been established by randomized evidence and remains influenced by patient selection, timing of initiation, institutional experience, and complication burden. Therefore, early surgery, delayed repair, percutaneous closure, and mechanical circulatory support should not be viewed as competing fixed strategies, but as components of an individualized management algorithm guided by hemodynamic status, defect anatomy, ventricular function, end-organ perfusion, and multidisciplinary Heart Team assessment.

## 5. Conclusions

Post-infarction ventricular septal defect remains a life-threatening condition requiring rapid diagnosis and individualized management. Right ventricular dysfunction plays a central role in clinical deterioration, particularly in inferior infarction. Early surgical repair is mandatory in hemodynamically unstable patients, while delayed strategies may be considered in selected stabilized cases. Mechanical circulatory support, especially VA-ECMO, represents a key adjunct in the management of postoperative cardiogenic shock, allowing ventricular recovery and improving survival. A multidisciplinary Heart Team approach remains essential for optimizing both short- and long-term outcomes.

## Data Availability

The raw data supporting the conclusions of this article will be made available by the authors on request.

## References

[1] Moore C.A., Nygaard T.W., Kaiser D.L., Cooper A.A., Gibson R.S. (1986). Postinfarction ventricular septal rupture: The importance of infarct location and right ventricular function in determining survival. Circulation.

[2] Crenshaw B.S., Granger C.B., Birnbaum Y., Pieper K.S., Morris D.C., Kleiman N.S., Vahanian A., Califf R.M., Topol E.J. (2000). Risk factors, angiographic patterns, and outcomes in patients with ventricular septal defect complicating acute myocardial infarction. GUSTO-I (Global Utilization of Streptokinase and TPA for Occluded Coronary Arteries) Trial Investigators. Circulation.

[3] Moreyra A.E., Huang M.S., Wilson A.C., Deng Y., Cosgrove N.M., Kostis J.B., MIDAS Study Group (MIDAS 13) (2010). Trends in incidence and mortality rates of ventricular septal rupture during acute myocardial infarction. Am. J. Cardiol..

[4] Papalexopoulou N., Young C.P., Attia R.Q. (2013). What is the best timing of surgery in patients with post-infarct ventricular septal rupture?. Interact. Cardiovasc. Thorac. Surg..

[5] Arnaoutakis G.J., Zhao Y., George T.J., Sciortino C.M., McCarthy P.M., Conte J.V. (2012). Surgical repair of ventricular septal defect after myocardial infarction: Outcomes from the Society of Thoracic Surgeons National Database. Ann. Thorac. Surg..

[6] Jones B.M., Kapadia S.R., Smedira N.G., Robich M., Tuzcu E.M., Menon V., Krishnaswamy A. (2014). Ventricular septal rupture complicating acute myocardial infarction: A contemporary review. Eur. Heart J..

[7] Ronco D., Ariza-Solé A., Kowalewski M., Matteucci M., Di Mauro M., López-de-Sá E., Ranucci M., Sionis A., Bonaros N., De Bonis M. (2023). The current clinical practice for management of post-infarction ventricular septal rupture: A European survey. Eur. Heart J. Open.

[8] David T.E., Armstrong S. (1998). Surgical repair of postinfarction ventricular septal defect by infarct exclusion. Semin. Thorac. Cardiovasc. Surg..

[9] Żbikowska K., Wróbel K. (2022). Mechanical Circulatory Support in Delayed Surgery of Post-Infarction Ventricular Septal Rupture in Patients in Cardiogenic Shock-A Review. J. Clin. Med..

[10] Schrage B., Ibrahim K., Loehn T., Werner N., Sinning J.M., Pappalardo F., Pieri M., Skurk C., Lauten A., Landmesser U. (2019). Impella Support for Acute Myocardial Infarction Complicated by Cardiogenic Shock. Circulation.

[11] Daggett W.M. (1978). Surgical management of ventricular septal defects complicating myocardial infarction. World J. Surg..

[12] Mashiko K., Ishii S., Naganuma H., Sakamoto H., Yagi H., Seo A., Mikawa H. (1999). Surgical repair of postinfarction ventricular septal defect: Modified Komeda-David procedure. Kyobu Geka.

[13] Schlotter F., de Waha S., Eitel I., Desch S., Fuernau G., Thiele H. (2016). Interventional post-myocardial infarction ventricular septal defect closure: A systematic review of current evidence. EuroIntervention.

[14] Calvert P.A., Cockburn J., Wynne D., Ludman P., Rana B.S., Northridge D., Mullen M.J., Malik I., Turner M., Khogali S. (2014). Percutaneous closure of postinfarction ventricular septal defect: In-hospital outcomes and long-term follow-up of UK experience. Circulation.

[15] Holzer R., Balzer D., Amin Z., Ruiz C.E., Feinstein J., Bass J., Vance M., Cao Q.-L., Hijazi Z.M. (2004). Transcatheter closure of postinfarction ventricular septal defects using the Amplatzer device. Catheter. Cardiovasc. Interv..

[16] Aramin M.A.S., Abuhashem S., Faris K.J., Omar B.M.M., Burhanuddin M., Teja P.S., Ibraheim M. (2024). Surgical closure versus transcatheter closure for ventricular septal defect post-infarction: A meta-analysis. Ann. Med. Surg..

[17] Matteucci M., Fina D., Jiritano F., Meani P., Kowalewski M., Lorusso R. (2022). Treatment strategies for post-infarction ventricular septal rupture: A meta-analysis. Eur. J. Cardiothorac. Surg..

[18] Ibanez B., James S., Agewall S., Antunes M.J., Bucciarelli-Ducci C., Bueno H., Caforio A.L.P., Crea F., Goudevenos J.A., Halvorsen S. (2018). 2017 ESC Guidelines for the management of acute myocardial infarction in patients presenting with ST-segment elevation: The Task Force for the management of acute myocardial infarction in patients presenting with ST-segment elevation of the European Society of Cardiology (ESC). Eur. Heart J..

[19] Thiele H., Zeymer U., Neumann F.J., Ferenc M., Olbrich H.G., Hausleiter J., Richardt G., Hennersdorf M., Empen K., Fuernau G. (2012). Intraaortic balloon support for myocardial infarction with cardiogenic shock. N. Engl. J. Med..

[20] Extracorporeal Life Support Organization (2021). ELSO Adult Cardiac Support Registry Report.

[21] Abrams D., Combes A., Brodie D. (2014). Extracorporeal membrane oxygenation in cardiopulmonary disease in adults. J. Am. Coll. Cardiol..

[22] Pappalardo F., Schulte C., Pieri M., Schrage B., Contri R., Soeffker G., Greco T., Lembo R., Müllerleile K., Colombo A. (2017). Concomitant implantation of Impella^®^ on top of veno-arterial extracorporeal membrane oxygenation may improve survival of patients with cardiogenic shock. Eur. J. Heart Fail..

[23] Meani P., Gelsomino S., Natour E., Johnson D.M., Rocca H.P., Pappalardo F., Bidar E., Makhoul M., Raffa G., Heuts S. (2017). Modalities and effects of left ventricle unloading on extracorporeal life support. Eur. J. Heart Fail..

[24] Modi S.P., Hong Y., Sicke M.M., Hess N.R., Klass W.J., Ziegler L.A., Rivosecchi R.M., Hickey G.W., Kaczorowski D.J., Ramanan R. (2023). Concomitant Use of VA-ECMO and Impella Support for Cardiogenic Shock. medRxiv.

[25] Varghese S., Ohlow M.A. (2019). Left ventricular free wall rupture in myocardial infarction: A retrospective analysis from a tertiary care center. Cardiol. Res..

[26] Matteucci M., Ferrarese S., Mantovani V., Corazzari C., Cappabianca G., Messina C., Garis S., Severgnini P., Lorusso R., Musazzi A. (2024). Surgical repair of left ventricular free-wall rupture complicating acute myocardial infarction: A systematic review. Front. Cardiovasc. Med..

